# Propranolol Use Among Healthcare Students in Saudi Arabia

**DOI:** 10.7759/cureus.48606

**Published:** 2023-11-10

**Authors:** Seham Aljahdali, Rahaf Badr, Maryam Alotaibi, Seham Alhelali, Ghodwah Abdullatif, Asim Alshanberi, Arwa Fairaq, Sahar M Elashmony, Alaa H Falemban, Safaa Alsanosi, Yosra Z Alhindi

**Affiliations:** 1 Pharmacy, Umm Al-Qura University, Makkah, SAU; 2 Family Medicine, Umm Al-Qura University, Makkah, SAU; 3 Pharmacology and Therapeutics, Umm Al-Qura University, Makkah, SAU; 4 Pharmacology and Toxicology, Umm Al-Qura University, Makkah, SAU; 5 Pharmacology and Toxicology, Faculty of Medicine, Umm Al-Qura University, Makkah, SAU

**Keywords:** saudi arabia, umm al-qura university, healthcare student, academic anxiety, propranolol

## Abstract

Background: The learning performance and overall health of students might be impacted by excessive academic stress. While the right amount of stress can help improve learning and performance, too much stress can harm one’s mental and physical health as well as academic performance. This research aims to assess the prevalence and use of the beta-blocker propranolol among healthcare students in Saudi Arabia.

Methods: A cross-sectional study was conducted among healthcare students at Umm Al-Qura University in Makkah, Saudi Arabia. The participants were sent an electronic questionnaire at random over three months, from June 10 to September 10, 2023. The data were analyzed using RStudio (version 4.2.2), and the categorized data were presented in frequencies and percentages. Fisher’s exact test was used to determine the factors associated with propranolol use. The results were reported as odds ratios (OR) and 95% confidence intervals (CI). A p-value of < 0.05 was considered statistically significant.

Results: The study comprised 582 participants, of whom (51.7%, n=301) fell within the age range of 24 to 26 years, (63.1%, n=367) were male, and (59.3%, n=345) were enrolled in the College of Medicine and Surgery. The majority of respondents (73.7%, n=28) reported that educational materials such as medical books were their primary source of information regarding the impact of beta-blockers on anxiety. Among those who used propranolol, over two-thirds (68.4%, n=26) had taken it before the Objective Structured Clinical Examination (OSCE). About a quarter of the participants (26.1%, n=151) believed that propranolol was being misused by healthcare students, and (21.3%, n=123) believed that the drug could enhance academic performance.

Conclusion: The primary motives for taking propranolol were to alleviate anxiety before OSCEs and enhance performance during presentations. The participants showed some understanding of the impact of propranolol. Nevertheless, it is imperative to impart knowledge to them about the potential hazards linked to the misuse of beta-blockers.

## Introduction

Anxiety attacks may occur from time to time. Many people fret about subjects such as health, education, finances, or family issues [1]. However, for those who suffer from anxiety disorders, anxiety persists and may even worsen over time. Anxiety symptoms affect daily activities like job performance, academic success, and interpersonal relationships [2]. Anxiety has received less attention from healthcare professionals and is commonly misunderstood and untreated in the general population. Consequently, the incidence of anxiety among medical students requires greater attention; students who suffer from anxiety problems tend to approach their studies passively, with little motivation, and perform poorly on exams and homework [3,4].

Hypochondriasis is a form of health anxiety disease also known as ‘medical student illness’. It develops in response to students’ misinterpretations of bodily sensations or symptoms associated with the disease they are learning about. Medical students are thought to be more susceptible to this illness than students in other fields [5,6]. These medical students often look for various stress-relieving approaches and methods. The most popular method for reducing anxiety associated with educational stress is the use of medications, such as beta-blockers like propranolol [7].

Propranolol is a non-cardioselective beta-blocker used to treat hypertension, pheochromocytoma, myocardial infarction, cardiac arrhythmias, angina pectoris, and hypertrophic cardiomyopathy [8]. Additionally, it is used to reduce the symptoms of sympathetic overactivity in the treatment of tremors, anxiety disorders, and hyperthyroidism. Other indications include the prevention of upper gastrointestinal bleeding and migraines in those with portal hypertension [9,10]. Propranolol, as mentioned above, is used for anxiety and may be used in posttraumatic stress disorder, social phobia, and specific phobias, such as dentaphobia. A meta-analysis study showed that propranolol is as effective as benzodiazepines in reducing panic disorder and posttraumatic stress disorder [9-11].

The psychological symptoms of anxiety, such as feeling tense, anxious, worried, or having a sensation of panic, are not treated by propranolol. Propranolol is used to decrease physical symptoms. It is helpful when patients have transient anxiety related to particular social situations, such as public speaking and performance, to stop sweating, shaking, rapid heart rate, rapid breathing, and other symptoms related to anxiety [12]. Studies have previously been conducted to examine the prevalence of propranolol use for anxiety management in various areas of Saudi Arabia and worldwide [13-15]. Nevertheless, there is no existing research on healthcare students in the Makkah region. Therefore, this study aims to assess the prevalence and use of the beta-blocker propranolol among healthcare students in Saudi Arabia.

## Materials and methods

Ethical approval

The study was approved by the Institutional Research Board of Umm Al-Qura University in Makkah, Saudi Arabia. The approval number is HAPO-02-K-012-2023-011389, under the Declaration of Helsinki.

Study design

A cross-sectional study was conducted among healthcare students at Umm Al-Qura University in Makkah, Saudi Arabia. They were randomly approached by sending them the electronic questionnaire over three months, from June 10 to September 10, 2023. The participants were informed about the aim of the study, and the decision to participate in the study was voluntary and free.

Questionnaire tool

The questionnaire was adapted and validated in both Arabic and English from published studies by Al-Mohrej et al. [13] and Al-Halimi et al. [6]. The questionnaire comprised three sections: the first section collected sociodemographic data, such as age, gender, the college of the healthcare student, academic year, grade point average (GPA), and questions about comorbidities such as hypertension, diabetes, or heart disease. The second section comprised four close-ended questions to assess the prevalence of propranolol use. The last section comprised 11 close-ended questions to assess the drug’s usage patterns.

Study populations (inclusion/exclusion criteria)

The selection criteria included students over the age of 18 who were pursuing healthcare-related degrees, such as pharmacy, medicine, surgery, dentistry, medical applied sciences, public health sciences, and nursing at Umm Al-Qura University. The study sample excluded participants below 18 years old, non-healthcare students, and healthcare students from other universities.

Sample size and data collection

Data were collected through an electronic questionnaire in English and Arabic.

The sample size was calculated using Slovin’s formula, with a population size of 635 Medical Students al Qassim University, Saudi Arabia, from a recently published study by Elghazaly A et al. (2023), with a confidence interval (CI) of 95% and a margin of error of 5% (17). The study was conducted using a Google Form template and distributed to the target population using social media (WhatsApp, Telegram, and Twitter). The data were gathered through a questionnaire and then entered into an Excel sheet for statistical analysis. Survey responses were collected anonymously; no private or identifying information from the participants was collected, and all responses were maintained with strict confidentiality and used only for research purposes.

Statistical analysis

The data were analyzed using RStudio (version 4.2.2), and the categorized data were presented in frequencies and percentages. A Fisher’s exact test with a simulated p-value (based on 2000 replicates) was used to determine the factors associated with propranolol use without a prescription among healthcare students. The variables that were significantly associated with propranolol use were included in a multivariate binary logistic regression model to identify independent predictors of propranolol use. The results were reported as odds ratios (OR) and 95% confidence intervals (CI). A p-value of < 0.005 was considered statistically significant.

## Results

Demographic and clinical characteristics

**Figure 1:**
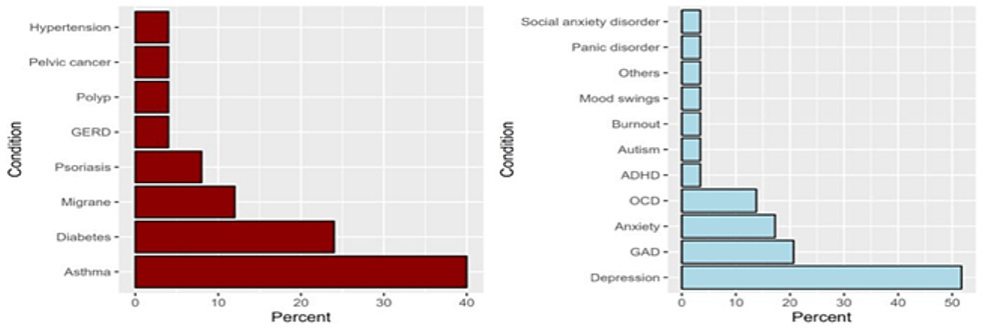
The proportion of chronic conditions among participants with comorbidities (A, n = 26) and the proportion of psychiatric disorders among participants with at least one self-reported psychiatric disorder (B, n = 29) (The data has been represented as N, %, p-value is considered significant (p<0.05) ADHD: Attention deficit hyperactivity disorders, OCD: Obsessive compulsive disorder, GAD: Generalized anxiety disorder, GERD: Gastroesophageal reflux disease

We received 638 responses to the questionnaire. However, we excluded 47 responses from those who disagreed with participating and nine responses from non-healthcare students at the university. Therefore, 582 responses were analyzed. The features of the respondents are recorded in Table 1. More than half of the respondents, aged 24 to 26 years (51.7%, n=301), were male (63.1%, n=367) and were studying at the Medical College (59.3%, n=345). Moreover, interns represented 54.5% (n=317) of the sample. More than one-third of the respondents (35.2%, n=205) consumed coffee while studying and preparing for exams, while 85.4% (n=497) slept for six to two hours per day during the exam weeks. Only 4.5% (n=27) of the sample had chronic conditions, predominantly asthma (40.0%, n=11) and diabetes (24.0%, n= 16; Figure 1A). Only 29 participants (5.0%, n=29) had been diagnosed with a psychiatric illness. The most common psychiatric disorders included depression (51.7%, n=15) and generalized anxiety disorder (20.7%, n=7; Figure 1B).

**Table 1:** Demographic and clinical characteristics of the participants (N=582) (The data has been represented as N, %, and a p-value is considered significant (p<0.05)

Parameter	Category	N (%)
Age	18 to 20	99 (17.0%)
	21 to 23	182 (31.3%)
	24 to 26	301 (51.7%)
Gender	Male	367 (63.1%)
	Female	215 (36.9%)
Colleg	Nursing	15 (2.6%)
	Medicine	345 (59.3%)
	Pharmacy	162 (27.8%)
	Medical Science	49 (8.4%)
	Dentistry	11 (1.9%)
Grade Point Average (GPA)	1.5 to 2.99	53 (9.1%)
	3 to 3.74	155 (26.6%)
	3.75 to 4	374 (64.3%)
Academic year	First year	37 (6.4%)
	Second year	57 (9.8%)
	Third year	31 (5.3%)
	Fourth year	70 (12.0%)
	Fifth year	65 (11.2%)
	Sixth year	5 (0.9%)
	Internship	317 (54.5%)
Do you exercise?	Yes	128 (22.0%)
	No	454 (78.0%)
Are you a smoker?	Yes	33 (5.7%)
	No	549 (94.3%)
Do you drink coffee while studying and preparing for exams?	Yes	205 (35.2%)
	No	377 (64.8%)
During exam week, how many hours do you usually sleep?	2-4 h	72 (12.4%)
	4-6 h	425 (73.0%)
	6-8 h	51 (8.8%)
	>8 h	34 (5.8%)
Have you been diagnosed with any chronic diseases?	Yes	26 (4.5%)
	No	556 (95.5%)
Have you been diagnosed with any psychiatric disorders?	Yes	29 (5.0%)
	No	553 (95.0%)
Do you have any type of anxiety diagnosed clinically?	Yes, I have social anxiety	31 (5.3%)
	Yes, I have generalized anxiety disorder	354 (60.8%)
	Yes, I have both	29 (5.0%)
	No	168 (28.9%)

Patterns of propranolol use

In general, propranolol was prescribed for medical conditions among 1.9% (n=11) of the participants. It was also used without a medical prescription for academic anxiety by 6.5% (n=2) of participants (Data is not presented in figures or tables here).

Focusing on the participants who used propranolol without a prescription (7.5%, n = 2, Table 2), self-prescription was prevalent among (60.5%, n= 20), of which (63.2%, n=24) took the medication at a dose of < 10 mg. Educational materials such as medical books and medical websites were the most common sources of knowledge about the effects of propranolol on anxiety. Around 15.8% (n=6) increased the dose without a physician’s instructions. More than half of the propranolol users had received the medication before the Objective Structured Clinical Examination (OSCE; 68.4%, n=26). Most users were aware of its side effects (73.7%, n=28), and approximately 23.7% (n=9) had experienced certain side effects. Hypotension was the most common side effect (66.7%, n=6), followed by fatigue (55.6%, n=5). Importantly, 36.8% (n=10) of the respondents indicated that they would recommend propranolol use to others, and 47.4% (n=18) of them declared that a friend or classmate had recommended the drug to them (Table 2).

**Table 2:** Characteristics of propranolol use among those who used the medication without a prescription (for academic-associated anxiety, n = 38) *The descriptive data was based on nine participants who had experienced side effects with propranolol use. The data has been represented as N, %, and a p-value is considered significant (p<0.05) OSCE: Objective structured clinical exam

Parameter	Category	N (%)
Sources of knowledge about the effects of beta blockers on anxiety	Education materials	28 (73.7%)
	Friends and classmates	12 (31.6%)
	Family	8 (21.1%)
	Internet surfing	5 (13.2%)
	Social media	9 (23.7%)
Who prescribed propranolol to you?	Yourself	27 (71%)
	Friend	6 (15.8%)
	Pharmacist in a community pharmacy	2 (5.3%)
	Prescribed by a physician	3 (7.9%)
What dosage of propranolol did you use to reduce anxiety?	<10 mg	24 (63.2%)
	10-20 mg	12 (31.6%)
	30-40 mg	2 (5.3%)
Have you increased the dosage without a physician’s instructions?	Yes	6 (15.8%)
	No	32 (84.2%)
When propranolol was used	Before the OSCE	26 (68.4%)
	Before exam presentations	18 (47.4%)
	Before written exams	5 (13.2%)
	During exams period	2 (5.3%)
	Other (study groups and social gathering)	6(15.8 %)
Awarness of the side effects of the propranolol	Yes	28 (73.7%)
	No	10 (26.3%)
Experienced any side effects	Yes	9 (23.7%)
	No	29 (76.3%)
Type of experienced side effects*	Fatigue	5 (55.6%)
	Shortness of breath	1 (11.1%)
	Cold extremities	4 (44.4%)
	Hypotension	6 (66.7%)
	Other	1 (11.1%)

Factors associated with propranolol use without prescriptions

Compared to their peers, participants aged 21 to 23 years exhibited significantly higher propranolol use without a prescription (17.6%, n=32 vs. 6.1%, n=6 among those aged 18 to 20 years and 0% among those aged 24 to 26 years, p < 0.05), as did females (13.5%, n=29 vs. 2.5%, n=9, among males, p < 0.05), smokers (36.4%, n=12 vs. 4.6%, n=21, among non-smokers, p < 0.05), those who drank coffee while studying and preparing for the exam (13.7%, n= 28 vs. 2.7%, n=10, p < 0.05), and those who exercised (13.3%, n= 17 vs. 4.6%, n= 21, p < 0.05). Propranolol use differed significantly according to sleep duration during exam weeks (p < 0.05), GPA (p < 0.05), academic year (p < 0.05), and the presence of an anxiety disorder (p < 0.05, Table 3). 

**Table 3:** Factors associated with propranolol use among healthcare students The data has been represented as N, %, and p-value is considered significant (p<0.05)

Parameter	Category		Using propranolol without prescription		p
			No, N = 544	Yes, N = 38	
Age		18 to 20	93 (93.9%)	6 (6.1%)	<0.05
		21 to 23	150 (82.4%)	32 (17.6%)	
		24 to 26	301 (100.0%)	0 (0.0%)	
Gender		Male	358 (97.5%)	9 (2.5%)	<0.05
		Female	186 (86.5%)	29 (13.5%)	
College		Nursing	13 (86.7%)	2 (13.3%)	0.406
		Medicine	321 (98.0%)	1 (2.0%)	
		Pharmacy	152 (93.8%)	10 (6.2%)	
		Medical Science	48 (93.0%)	24 (7.0%)	
		Dentistry	10 (90.9%)	1 (9.1%)	
Grade Point Average (GPA)		1.5 to 2.99	38 (71.7%)	15 (28.3%)	<0.05
		3 to 3.74	138 (89.0%)	17 (11.0%)	
		3.75 to 4	368 (98.4%)	6 (1.6%)	
Academic year		First year	37 (100.0%)	0 (0.0%)	<0.05
		Second year	52 (91.2%)	5 (8.8%)	
		Third year	30 (96.8%)	1 (3.2%)	
		Fourth year	60 (85.7%)	10 (14.3%)	
		Fifth year	48 (73.8%)	17 (26.2%)	
		Sixth year	1 (20.0%)	4 (80.0%)	
		Internship	316 (99.7%)	1 (0.3%)	
Do you exercise?		No	433 (95.4%)	21 (4.6%)	<0.05
		Yes	111 (86.7%)	17 (13.3%)	
Are a you smoker?		No	523 (95.3%)	26 (4.7%)	<0.05
		Yes	21 (63.6%)	12 (36.4%)	
Do you drink coffee while studying and preparing for exams?		No	367 (97.3%)	10 (2.7%)	<0.05
		Yes	177 (86.3%)	28 (13.7%)	
During exam week, how many hours do you usually sleep?		2-4 h	69 (95.8%)	3 (4.2%)	<0.005
		4-6 h	405 (95.3%)	20 (4.7%)	
		6-8 h	40 (78.4%)	11 (21.6%)	
		>8 h	30 (88.2%)	4 (11.8%)	
Have you been diagnosed with any chronic diseases?		No	518 (93.2%)	38 (6.8%)	0.403
		Yes	26 (100.0%)	0 (0.0%)	
Have you been diagnosed with any psychiatric disorder?		No	519 (93.9%)	34 (6.1%)	0.113
		Yes	25 (86.2%)	4 (13.8%)	
Do you have any type of anxiety?		No	151 (89.9%)	17 (10.1%)	<0.005
		Yes, I have social anxiety	27 (87.1%)	4 (12.9%)	
		Yes, I have generalized anxiety disorder	343 (96.9%)	11 (3.1%)	
		Yes, I have both	23 (79.3%)	6 (20.7%)	

Predictors of propranolol use

In this analysis, we incorporated the significantly associated variables from the association analysis, including participants’ age, gender, GPA, academic year, performance of exercise, smoking status, coffee consumption while studying, the number of hours slept per week during exam periods, and the presence of any type of anxiety. No influential outliers were found in the model. However, we excluded one variable (academic year) because it showed a risk of multicollinearity (a variance inflation factor of 13.6). The results showed that propranolol use was significantly predicted by being aged 21 to 23 years (OR = 3.7, 95% CI, 1.3 to 12.9, p = 0.025), being female (OR = 3.2, 95% CI, 1.1 to 10.1, p = 0.039), having a GPA of 1.5 to 2.99 (OR = 6.2, 95% CI, 1.7 to 26.2, p = 0.009), and being a smoker (OR = 4.4, 95% CI, 1.5 to 12.7, p = 0.006, Table 4).

**Table 4:** Results of the regression analysis for the predictors of propranolol use among healthcare students OR: odds ratio; NA: non-available because the variable had one zero frequency The data has been represented as OR, CI 95%, and a p-value is considered significant (p<0.05)

Parameter	Category	OR	95% CI	p
Age	18 to 20	Ref	Ref	
	21 to 23	3.73	1.27, 12.9	0.025
	24 to 26	NA	NA	0.987
Gender	Male	Ref	Ref	
	Female	3.17	1.11, 10.1	0.039
GPA	3.75 to 4	Ref	Ref	
	3 to 3.74	1.5	0.49, 5.27	0.495
	1.5 to 2.99	6.21	1.67, 26.2	0.009
Do you exercise?	No	Ref	Ref	
	Yes	1.84	0.79, 4.43	0.163
Are you a smoker?	No	Ref	Ref	
	Yes	4.39	1.51, 12.7	0.006
Do you drink coffee while studying and preparing for exams?	No	Ref	Ref	
	Yes	0.87	0.35, 2.28	0.767
During exam week, how many hours do you usually sleep?	>8 h	Ref	Ref	
	6-8 h	1.76	0.43, 8.58	0.451
	4-6 h	0.53	0.13, 2.51	0.388
	2-4 h	0.23	0.04, 1.37	0.103
Do you have any type of anxiety?	No	Ref	Ref	
	Yes, I have social anxiety	0.82	0.19, 2.87	0.769
	Yes, I have generalized anxiety disorder	1.05	0.34, 3.05	0.924
	Yes, I have both	1.64	0.45, 5.38	0.430

Out of the whole study sample (n = 582), approximately one-quarter of the participants thought that there was propranolol misuse among healthcare students (26.1%, n=152) and that the medication could improve academic performance (21.3%, n=124).

## Discussion

This cross-sectional study was carried out among Umm Al-Qura University healthcare students from different colleges using convenience sampling. A total of 582 out of 638 students were included in the study. Earlier research has been conducted on the prevalence of propranolol usage for anxiety reduction in diverse regions of Saudi Arabia and globally. However, to the best of our knowledge, this is the first investigation of healthcare students in the Makkah region.

Anxiety is a prevalent psychological difficulty, particularly among healthcare students, who experience greater stress and pressure during their studies and clinical training. These challenges can have an adverse effect on their mental health, leading to various psychological problems such as anxiety and depression. Within this group, it is suspected that propranolol, which is known to reduce anxiety, may be misused [16-20].

The misuse of propranolol among healthcare students differs between colleges in Saudi Arabia. Our study revealed that the prevalence of propranolol misuse was very low. According to our results, only 6.5% (n=38) of healthcare students used propranolol to control anxiety without a prescription. The prevalence at King Faisal University in Al-Ahsa was the lowest, with only 0.79% of the participants claiming to use propranolol for academic anxiety [16,21]. However, the prevalence of propranolol was higher at King Saud bin Abdulaziz University for Health Sciences in Riyadh, where the use of propranolol among medical and dental students was 30% [16]. The highest prevalence of propranolol misuse was found at King Abdulaziz University in Jeddah, with a prevalence of 40% [16]. Our results demonstrate that the prevalence of propranolol use to reduce academic anxiety is higher among females. These results were consistent with those of Al-Halimi, who found that all propranolol users in that study were female [16]. There is a potential explanation for this finding, which is that females generally experience more psychological distress than males in the general population and that there are differences in emotional experiences and expressions between genders. The specific causes of this gender difference in anxiety prevalence are not completely understood, but possible factors that may play a role include hormonal changes, societal and cultural influences, and variations in brain chemistry [22,23]. However, these results contradict Al-Mohrej’s findings, in which 78% of propranolol users were male [13].

The prevalence of propranolol use to reduce academic anxiety was higher among senior students, which may be because healthcare students in their last year of college may have more concerns regarding achievement and have higher academic expectations [7]. Our results are consistent with Abukhalaf et al., who showed that junior students used beta-blockers less often than senior students [24]. In addition, the prevalence of propranolol use to reduce academic anxiety was higher among students with lower GPAs, between 1.5 and 2.99. A possible explanation for this result may be that anxiety is a significant predictor of low academic performance, and students with anxiety are unable to perform to their best abilities [25]. The findings from our research indicate that most of the medical students (68.4%, n=26) who used propranolol took it before their OSCE (Objective Structured Clinical Exam), with (18%, n=6) using it before oral presentations that had a social component. This is consistent with a study conducted at King Saud bin Abdulaziz University for Health Sciences in Jeddah, where it was found that 70.6% of propranolol users took the drug before their OSCEs to alleviate anxiety [26]. Similarly, another study found that heightened levels of anxiety and stress have been observed to reduce the effectiveness and confidence of students taking the OSCE test [27]. Interestingly, over half of the students who used propranolol in our study were in their internship years. Their usage might be attributed to the stressful nature and demanding hours of their work programs.

The prevalence of self-reported propranolol use among healthcare students at Umm Al-Qura University was 8.4% (n=3), of which 6.5% (n=2) were users without medical prescriptions. However, comparing our findings with previous studies conducted in Saudi Arabia, it was found that self-medication was prevalent among 60.5% of the participants and that 63.2% of them had taken propranolol at a dose of < 10 mg [13,21,24,26]. The highest reported dose was 40 mg, which was close to the highest reported dose in our results [28]. Only 15.8% (n=6) of participants at Umm Al-Qura University increased their dosage without a physician’s instructions; according to Alkhatabi et al., 29.4% of their participants increased their dosage without a physician’s instructions [26]. A possible explanation for this could be that pharmacies are conveniently located; some students may choose to use medications without a doctor’s prescription, and some pharmacies supply propranolol without a prescription [24].

As the study was conducted among healthcare students, most participants were aware of propranolol’s side effects (73.7%, n=28). This was consistent with previous studies conducted in Saudi Arabia [13,21,26], which indicated that these students were aware of the side effects, including hypotension, fatigue, and shortness of breath. Although caffeine consumption improves performance and mental alertness, evidence indicates that the consumption of high levels of caffeine can be related to increased anxiety levels [29]. According to our results, the participants who drank coffee while studying and preparing for their exams had greater levels of anxiety and were likelier to use propranolol than students who did not consume coffee. Similarly, smoking was one of the factors that increased propranolol use to reduce academic anxiety, and there is some evidence that smoking increases behavioral problems, including stress and anxiety, among healthcare students [18-20].

However, the sample size of participants using propranolol is too small to do further analysis.

Our study revealed that propranolol users who practiced exercise experienced a 13.3% (n=2) reduction in anxiety levels, which was significantly higher than those who relied solely on propranolol to manage their anxiety (4.6%, n=1). This aligns with a previous study that investigated the impact of exercise on anxiety and found that individuals who regularly engaged in physical activity had fewer symptoms of depression and anxiety. These findings lend support to van Minnen et al.’s suggestion that exercising may serve as a protective measure against the development of mental health disorders [30-32].

The current study was limited by the study design, as data were obtained by self-reporting; hence, the responses to the questionnaire may not reflect the actual attitudes or behaviors of the participants. Another limitation is that we did not include the views of participants who did not use social media. Despite these constraints, this study is a unique addition to the existing literature on the use of propranolol by healthcare students in the Makkah region of Saudi Arabia.

Moreover, the sample size of the participants using propranolol is small, so multivariate analysis was not done.

## Conclusions

The prevalence of propranolol usage for anxiety reduction among healthcare students at Umm Al-Qura University was found to be quite low. Female students exhibited higher rates of anxiety than their male counterparts, and the primary reason for taking propranolol was academic anxiety during the OSCE exam. However, it is crucial to educate medical students about the potential risks of improper use of beta-blockers to minimize their usage and prevent any potential negative consequences associated with the medication.
